# Supplementary material for: Neuropathological interpretation of stimulated Raman histology images of brain and spine tumors: part B
Source: Neurosurg Rev. 2021 Dec 10;45(2):1721–9. doi: 10.1007/s10143-021-01711-1 (PMC8976804; doi:10.1007/s10143-021-01711-1)
Supplement: Supplementary file 1 — Supplementary file1 (DOCX 3249 KB) [file 10143_2021_1711_MOESM1_ESM.docx]

**Neuropathological interpretation of Stimulated Raman Histology images of brain and spine tumors - Part B**

Jakob Straehle^1^, Daniel Erny^2^, Nicolas Neidert^1,3^, Dieter Henrik Heiland^1,3,4,5,15^, Amir El Rahal^1^, Vlad Sacalean^1,3^*,* David Steybe^6^, Rainer Schmelzeisen^6,15^, Andreas Vlachos^7,8,9,15^, Boris Mizaikoff^10,11^, Peter Christoph Reinacher^12,13,15^, Volker Arnd Coenen^12,15^, Marco Prinz^2,14,8,15^, Jürgen Beck^1,4,8,15^ and Oliver Schnell^1,15^

^1^ Department of Neurosurgery, Medical Center, University of Freiburg, Freiburg, Germany

^2^ Institute of Neuropathology, Faculty of Medicine, University of Freiburg, Freiburg, Germany

^3^ Microenvironment and Immunology Research Laboratory, Medical Center, University of Freiburg, Freiburg, Germany

^4^ Comprehensive Cancer Center Freiburg (CCCF), Faculty of Medicine and Medical Center, University of Freiburg, Freiburg, Germany.

^5^ German Cancer Consortium (DKTK), partner site Freiburg, Freiburg, Germany

^6^ Department of Oral and Maxillofacial Surgery, Medical Center, University of Freiburg, Freiburg, Germany

^7^ Department of Neuroanatomy, Institute of Anatomy and Cell Biology, Faculty of Medicine, University of Freiburg, Freiburg, Germany

^8^ Center for Basics in NeuroModulation (NeuroModulBasics), Faculty of Medicine, University of Freiburg, Freiburg, Germany

^9^ Center Brain Links Brain Tools, University of Freiburg, Freiburg, Germany

^10^ Institute of Analytical and Bioanalytical Chemistry, Ulm University, Ulm, Germany

^11^ Hahn-Schickard Institute for Microanalysis Systems, Ulm, Germany

^12^ Department of Stereotactic and Functional Neurosurgery, Medical Center, University of Freiburg, Freiburg, Germany

^13^ Fraunhofer Institute for Laser Technology (ILT), Aachen, Germany

^14^ Signalling Research Centres BIOSS and CIBSS, University of Freiburg, Freiburg, Germany.

^15^ Medical Faculty of Freiburg University, Freiburg, Germany

# Corresponding Author

Prof. Dr. med. Oliver Schnell, MHBA

Department of Neurosurgery

Medical Center - University of Freiburg

Freiburg, Germany

Breisacher Str. 64

79106 Freiburg

Tel.: +49 761 270 50070

Fax: +49 761 270 51020

oliver.schnell@uniklinik-freiburg.de

ORCID-ID: 0000-0002-1284-1427

# Abstract

**Objective:** Intraoperative histopathological examinations are routinely performed to provide neurosurgeons with information about the entity of tumor tissue. Here, we quantified the neuropathological interpretability of Stimulated Raman Histology (SRH) acquired using a Raman laser imaging system in a routine clinical setting without any specialized training or prior experience.

**Methods:** Stimulated Raman Scattering microscopy was performed on 117 samples of pathological tissue from 73 cases of brain and spine tumor surgeries. A board-certified neuropathologist - novice in the interpretation of SRH - assessed image quality by scoring subjective tumor infiltration and stated a diagnosis based on the SRH images. The diagnostic accuracy was determined by comparison to frozen Hematoxylin-Eosin (H&E)-stained sections and the ground truth defined as the definitive neuropathological diagnosis.

**Results:** The overall SRH imaging quality was rated high with the detection of tumor cells classified as inconclusive in only 4.2% of all images. The accuracy of neuropathological diagnosis based on SRH images was 87.7% and was non-inferior to the current standard of fast frozen H&E-stained sections (87.3 vs. 88.9%, p=0.783). We found a substantial diagnostic correlation between SRH based neuropathological diagnosis and H&E-stained frozen sections (κ=0.8).

**Conclusion:** The interpretability of intraoperative SRH imaging was demonstrated to be equivalent to the current standard method of H&E-stained frozen sections. Further research using this label-free innovative alternative vs. conventional staining is required to determine to which extent SRH-based intraoperative decision-making can be streamlined in order to facilitate the advancement of surgical neurooncology.

# Keywords

Stimulated Raman Histology (SRH), neuropathology, diagnostic accuracy, neurosurgery, NIO, H&E-stained frozen section

# Statements and Declarations

# Funding

J.S. received funding from the Berta-Ottenstein-Programme for Clinician Scientists, Faculty of Medicine, University of Freiburg, Germany. D.E. received funding from the Berta-Ottenstein-Programme for advanced Clinician Scientists, Faculty of Medicine, University of Freiburg, Germany. D.H.H. is funded by the Else Kröner-Fresenius Foundation. The work is part of the MEPHISTO project (PI: DHH et al.), funded by BMBF (German Ministry of Education and Research) (project number: 031L0260B). A.E.R received funding by the Nuovo-Soldati Foundation.

# Conflicts of interests

On behalf of all authors, the corresponding author states that there is no conflict of interest.

# Availability of data and material

Not applicable

# Code availability

Not applicable

# Ethics approval

Tissue samples were collected and imaged in accordance with the guidelines of the biobank at the Department of Neurosurgery, Medical Center University of Freiburg with the approval of the local ethics committee of the University of Freiburg (protocol 5565/15) and with written informed patient consent.

# Consent to participate

Written informed consent from participants is available

# Consent to publication

Consent for publication from participants is available

# Authors’ contributions

J.S., N.N., J.B. and O.S. designed the study; Funding: M.P., D.H.H., R.S., A.V. and J.B.; Data acquisition: J.S., N.N., D.S., A.E.R. V.S. and D.E.; P.R. and V.A.C. provided samples and infrastructure; Data analysis: J.S., D.H.H. and D.E.; Manuscript writing: J.S., J.B., D.H.H., B.M. and O.S. with contributions of all authors.

# Abbreviations

CI: confidence interval, CNS: central nervous system, FOV: field of view, GBM: glioblastoma, H&E: hematoxylin and eosin, HGG: high-grade glioma, IHC: immunohistochemistry, LGG: low-grade glioma, NSCLC: non-small cell lung cancer, SRH: Stimulated Raman Histology

# Introduction

State-of-the-art intraoperative histopathological diagnosis in a neurosurgical setting is routinely performed using fast frozen section and H&E-staining. This technique is limited by the amount of samples that can be processed in real-time in a routine clinical setting, as for a single sample there is a delay of 15-30 min from tissue removal to histopathological diagnosis. Therefore, an exhaustive histopathological sampling of tumor margins to assist neurosurgical decision-making in real-time is currently not available during routine tumor resections. Decisions on the extent of resection rather rely on subjective criteria such as visual and haptic impressions of the neurosurgeon, 5-Aminolaevulenic Acid (5-ALA) mediated fluorescence [23], intraoperative MRI [21], ultrasound, neuromonitoring [20] or neuronavigation.

A technological breakthrough [6, 7] leading to the development of a mobile stand-alone fiber-laser-based stimulated Raman scattering microscope (NIO Invenio Imaging Inc.) enables to perform Stimulated Raman Histology (SRH) with minimal delay (~3 min) label-free (i.e., without additional staining) directly in the operating theater [16].

Compared to conventional H&E-staining, SRH has several advantages such as the ease of use, the time of data processing and the digital nature of the obtained images. Digitalization allows remote consultations and image processing using automated classifiers and machine learning routines [9].

At the core of the novel application of SRH is the fundamental understanding of the obtained SRH images. Despite obvious analogies between SRH images and H&E-stained sections there are also differences. Firstly, tissue specimen used in SRH are squash preparations where characteristic cytoarchitectonic features of the tissue may be disrupted. Secondly, SRH images highlight cellular features - mainly tumoral and glia fibers - but also putative axons that are usually not evident on conventional H&E stains. The incorporation of intraoperative SRH imaging into clinical routine requires a close interdisciplinary exchange, whereby neurosurgeons and neuropathologists potentially with the support of expert vibrational spectroscopists become familiar with these new imaging modalities.

Following the introduction of intraoperative SRH imaging at our institution, we have tested the applicability of this technique in a routine clinical setting (Neidert et al., 2021). The aim of the current study was to (1) quantify the interpretability of SRH images and to (2) evaluate the diagnostic accuracy of SRH compared to the current standard.

# Materials and Methods

**Tissue acquisition and preparation**

Tissue samples were collected and imaged in accordance with the guidelines of the biobank at the Department of Neurosurgery, Medical Center University of Freiburg with the approval of the local ethics committee of the University of Freiburg (protocol 5565/15) and with written informed patient consent. 117 samples of putative pathological tissue from 73 consecutive surgical cases of brain, spine and peripheral tumors (1.6 ± 0.9 range from 1 to 5 samples/case) were investigated using stimulated Raman scattering microscopy. Surgeries included tumor resections (n=67) and stereotactic biopsies (n=6). The usual surgical routine was neither disrupted nor influenced.

Samples for SRH images were collected from the adjacent areas of samples extracted for conventional routine diagnostics. SRH was performed as described in detail in the accompanying study (Neidert et al., 2021). In all cases, pathological tissue was sampled and subject to conventional neuropathological processing (minimal time to diagnosis 2 days). According to our institution’s standard of care, in 63 of 73 cases additional tissue was sampled and processed for intraoperative neuropathological diagnostic using fast frozen H&E-stained sections.

**Sample processing and SRH imaging**

Small (1-3 mm edge size) samples were compressed to a thickness of 230 µm on a sample holder designed for use in the Raman imaging system (NIO Slide; NIO Invenio Imaging Inc.). SRH images were generated using a fiber-laser-based Stimulated Raman Scattering microscope (NIO Laser Imaging System, Invenio Imaging Inc, Santa Clara, CA, USA). Briefly, the raw image data consisted of Raman shifts at wavenumbers 2845 cm^–1^ and 2940 cm^–1^, respectively, corresponding to the vibrational frequencies of C-H_2_ bonds (primarily characteristic for lipids) and C-H_3_ bonds (primarily characteristic for proteins and DNA), respectively. The raw images were generated as sequential line scans of 1000 pixels in width at an imaging depth of 10 µm below the coverslip. The nominal pixel size was 467 nm. The images were automatically stitched and converted to SRH (i.e., ‘virtual H&E’) images via subtraction and the use of a proprietary lookup table (see ref. [16]), which is part of the NIO software package (version 1.5.0). In total, 309 SRH images with an average of 4.2 ± 2.3 (range 1 to 12) images per case were generated. In 237 of 309 images, the size of the field of view was nominally set to 2x2 mm resulting in an effective field-of-view of 3.06 mm^2^. The average image size was 3.7 ± 2.4 ranging from 0.2 to 19.4 mm^2^. Images were exported in DICOM format. For ease-of-use, SRH images were converted to .tif format using custom written scripts (Matlab R2020a).

**Criteria for the neuropathological assessment of SRH images**

The SRH images were presented to a board-certified neuropathologist (D.E.), who had no prior experience in the interpretation of SRH. To evaluate SRH image quality, each image of putative pathological tissue was classified with respect to the assessability of tumor infiltration using a ranked score: 1) infiltration is certain or can be certainly excluded, 2) possible infiltration and 3) inconclusive, where assessment of infiltration is not possible. Next, the medical information including age, tumor location and an anonymized brief medical history otherwise identical to the information available at the time of the conventional neuropathological consultation was revealed. Then, based on all SRH images per case the neuropathologist stated a diagnosis. As a guideline the following categories were an optional diagnosis: high-grade glioma, low-grade glioma, metastatic brain or spinal tumor, meningioma, CNS lymphoma, pituitary adenoma, sub-/ependymoma, sarcoma, schwannoma, necrosis, reactive gliosis, ganglioglioma, hemangioblastoma, colloid cyst, epidermoid, unclear or other tumors. The written SRH and H&E-based diagnosis were then classified into the final categories (n=14) by a referee (D.H.H.).

**Statistical analysis**

Group comparisons were performed using chi-squared testing. Significance was defined as an alpha-level <5%. Precision and recall between SRH or H&E-stained frozen section diagnosis and the ground truth (i.e., definite diagnosis) were calculated using the F measurement function in the FlowSOM package in R (RStudio Version 1.4.1106). F-statistic testing was performed using linear models function in R. The inter-rater correlation was calculated using Cohen’s kappa coefficient [2] using the psych package in R.

# Results

73 consecutive neurosurgical cases of brain, spine and peripheral tumors (Fig. 1a) were investigated using SRH imaging. The mean patient age was 55.9 ± 18.1 years with 39 female and 34 male patients. The majority of tumors originated from the brain (n=63) with high-grade gliomas (HGG, n=17) and brain metastasis (n=13) forming the largest groups (Fig. 1a). In this study, we defined HGG as WHO grade III and IV gliomas, and low-grade gliomas (LGG) as WHO grade I and II. The medical history was classified into 3 groups: 1) novel primary tumors (n=39), 2) recurrent disease (n = 16) and 3) primary metastatic disease with known potential origin outside the CNS (n=18) (Fig. 1b). While most cases of brain tumors (37/63) were primary, the majority of spine tumors (8/10) were metastatic with a known primary tumor (Fig. 1b). Examples of SRH images of the 6 largest categories of tumors (cf. Fig. 2j, 3b) are shown in Fig. 1c-h.

**Assessment of tumor infiltration in SRH images**

To evaluate the interpretability of the obtained SRH images (n=309) of putative pathological tissue, we established a ranked score based on the subjective confidence of a neuropathologist, who was novice in the interpretation of SRH images. The presence or absence of tumorous cells was assessed regardless of the underlying pathology. The score comprised 3 classes of tumor infiltration: *Class 1)* certain infiltration (yes/no); *Class 2)* possible infiltration; *Class 3)* inconclusive (Fig. 2a-f). Overall, 67.6% of SRH images were classified as class 1, 28.2% as class 2 and 4.2% as inconclusive class 3, respectively (Fig. 2g). The majority of SRH images (10 of 13) classified as class 3 were reported within the first 10 cases that were analyzed. They included necrosis (n=2), fibrous tissue (case of spinal metastasis, n=1), white matter (n=2, Suppl. Fig. 1), cases of diffuse infiltrating tumor (LGG and neuroblastoma, n=7) and SRH images taken close to the sample border (sampling error, n=1).

Stratification of subjective interpretability of SRH images by sample origin (Fig. 2h) showed a non-significant difference in class 1 confidence in brain vs. spinal tumors (66.92 vs. 70.73%, p=0.62 chi-squared). Prior treatment such as radio- and/or chemotherapy decreased the interpretability of SRH images (Fig. 2i). In cases of recurrent disease (n=16) vs. primary tumor cases (n=57, group 1 and 3, Fig. 1b) the class 1 confidence of tumor infiltration was significantly reduced (56.0 vs. 71.4%, p=0.013 chi-squared). Stratification of subjective interpretability of SRH into the six largest tumor categories (cf. Fig. 1c-h) revealed above average certainty of tumor infiltration in the case of pituitary adenoma, meningioma and metastasis, while below average certainty was observed in LGG and reactive gliosis.

**Accuracy of neuropathological diagnosis based on SRH images**

The neuropathologist stated a diagnosis based on all available SRH images/case and the medical history. In 2 out of 73 cases (i.e., primary epidermoid in the 4^th^ ventricle and recurrent GBM in right temporal lobe) a diagnosis was not possible. Using the final neuropathological report as a ground truth, the overall accuracy of the diagnosis based on SRH imaging was 87.7% (64 of 73 cases, Fig. 3a). The diagnostic accuracy based on SRH images was below average for reactive gliosis, while there was a 100% accuracy in LGGs, pituitary adenomas and brain or spine metastasis (Fig. 3b). For HGGs and meningiomas the diagnostic accuracy using SRH images was 88.9% and 90.9%, respectively.

**Comparison of diagnosis based on SRH images to fast frozen H&E-stained sections**

A comparison of the SRH based diagnosis to the current standard in neuropathological diagnosis based on fast frozen H&E-stained sections was performed in 63 cases (Fig. 3c,e). The accuracy of diagnosis based on SRH was 87.3% and 88.9% based on H&E-stained sections, respectively. The difference was not significant (p=0.783, chi-squared) demonstrating that the diagnostic accuracy of SRH is en par with H&E-stained fast frozen sections, which was previously reported [4]. The correlation between the diagnosis based on SRH vs. H&E-stained sections was determined using Cohen’s Kappa [2], and was indeed high at κ=0.80 ([0.70 to 0.91] 95% CI). The precision and recall of the diagnosis based on SRH images compared to the ground truth was 0.94 and 0.90, respectively (p=2.5E-10) with a diagnostic of correlation of κ = 0.84 ([0.75, 0.94] 95% CI, unweighted Cohen’s Kappa). Similarly, the precision and recall for the diagnosis based on H&E-stained sections was 0.96 and 0.89 (p=2.2E-16) with a diagnostic correlation of κ=0.86 ([0.77, 0.96] 95% CI).

**Estimation of bias to diagnostic accuracy**

To estimate bias via the knowledge of the medical history on the neuropathological decision making, the accuracy of primary tumors (i.e., without any previous neuropathological reports) was compared to the groups of recurrent disease and metastatic tumors with a known primary disease outside the CNS (group 2 and 3 Fig. 1c). The diagnostic accuracy in the non-primary group was higher, although not reaching statistical significance (84.6 vs. 91.2%, p=0.395, chi-squared test, Fig. 3d).

# Discussion

We report the first experience at our institution with the neuropathological interpretation of ex-vivo SRH images, conducted in a routine clinical scenario without any specialized training. Compared to other novel tools for intraoperative histological examination in-vivo such as fluorescein-assisted confocal laser endomicroscopy [8, 14] and hand-held (multimodal) Raman spectroscopy probes [3, 10], the ex-vivo SRH approach described here is limited by the fact that the tissue must be removed. Despite the significant speedup of SRH compared to conventional histopathology [4], the residual delay and the inability to process samples in parallel hinders repeated arbitrary sampling. Rather, a resection strategy including the sequential histopathological examination of multiple locations of interest is required (see below).

**Assessment of tumor infiltration in putative pathological samples**

In 67.6% of all images we found a high confidence in the assessment of tumor infiltration by a neuropathologist, who was unfamiliar with the interpretation of SRH images. Post-hoc analysis revealed that 6 samples contained cortex adjacent to the tumor; therefore, the class of “certain infiltration” also encompassed certain non-infiltrated (i.e., putative healthy) tissue (Fig. 2a,b). We observed a steep learning curve where the majority of images labeled as inconclusive (4.2%, Fig. 2g), were reported within the first 10 cases of unsupervised annotation.

Neuropathological diagnosis based on frozen sections of brain tumors in the case of re-operations were shown to have a significantly lower diagnostic accuracy compared to primary brain tumors (82 vs. 92%, respectively) [24]. As expected, we found the interpretability of brain tumor infiltration to be significantly reduced in cases of recurrent disease (Fig. 2i), which made up 22% of all cases in this study (cf. Fig. 1b).

**Diagnostic accuracy of SRH based neuropathological diagnosis**

The accuracy of the SRH-based neuropathological diagnosis from the first samples analyzed at our institution (including the very first) was 87.7% (Fig. 3a), which was close to the lower bound of the range of diagnostic accuracy (89-98%) reported for intraoperative frozen section neuropathological diagnosis [15, 24, 25]. It should be noted that the investigators had no prior experience with sample preparation specific to Raman imaging or SRH image interpretation. We expect that the diagnostic accuracy will further increase following ongoing training and the establishment of a standardized protocol.

The SRH-based diagnosis was most accurate for solid primary tumors (e.g., metastasis, meningioma, pituitary adenoma). Misclassifications occurred in cases of recurrent pre-treated tumors, reactive gliotic tissue and HGGs which also difficult to interpret in conventional H&E-stained tissues (Fig. 3e). Sampling errors were reported to be another major source of error in the conventional intraoperative neuropathological diagnosis [15]. In this study, spatially similar but not identical samples were processed in parallel for SRH and H&E. It is possible that distinct parts of the tumor were submitted to either method, yet not to the other. Furthermore, only parts of the samples were imaged, which may have led to undersampling of diagnostic features in SRH [16]. This is especially relevant for heterogeneous tumors (e.g. GBM), where necrotic and solid parts are interleaved.

**Non-inferiority of SRH diagnosis vs. H&E-stained frozen section**

As previously reported in a prospective trial by Eichberg et al. where SRH and conventional histology had a similar diagnostic accuracy [4], we found a non-inferiority of the pathological diagnosis based on SRH images compared to conventional fast frozen H&E-based images (87.3 vs. 88.9%, Fig 3c). The diagnostic correlation between SRH and the ground truth was κ=0.84, which is similar to the previously reported value of κ=0.83 [4]. The diagnostic correlation between SRH and H&E-stained sections reported here (κ=0.8) is lower than the near-perfect values reported in [16] (κ= 0.89–0.92), but is still considered to be substantial [12]. Recently, Pekmezci et al. [7] showed for resection borders of IDH mutated glioma that the gold standard of immunohistochemistry vs. SRH detected residual tumor at a similar rate (56% vs 49%).

We anticipate that neurosurgeons, neuropathologists and potentially neuroradiologists along with radiotherapists will in future become more familiar with the interpretation of SRH images, which will yield even more accurate interpretations. In addition, it is anticipated that appropriate deep learning / machine learning strategies [9] will augment the interpretation and classification of the obtained SRH images and further reduce the time-to-result. The present study provides a quantitative impression of the scenario neurosurgeons and neuropathologists will face when applying advanced imaging technologies in routine clinical settings.

**Workflow for the exploration of SRH-based neurosurgical decision making**

The first positive results reported here together with promising results attesting the diagnostic accuracy of SRH-based imaging similar to the current standard [4, 16, 17] lead us to conclude, - comparable to previous reports [11, 17] - that intraoperative SRH may be an adequate technique for histopathological-based surgical decision making close to real-time. Modern neuropathological diagnosis relies on a multitude of molecular markers far beyond a mere morphological description [1, 13]. It is therefore worth noting that intraoperative SRH is not intended to replace conventional pathological techniques, but rather work in parallel as a complementary tool in scenarios where a timely feedback is of the essence.

In a possible future surgical scenario of a brain tumor resection (Fig. 4), after exposure of the skull a frameless stereotactic biopsy [19] of the tumor at several points along a single trajectory via a burr hole centered on the planned craniotomy is performed (duration 10-20 min, Fig. 4b). The correlated samples are then processed in parallel using SRH and conventional methods using fast frozen sections (duration ~15-30 min) validating the SRH images at multiple locations. Performing the biopsy through a burr hole yields the best possible correlation with neuronavigation by minimizing brain shift as well as loss of accuracy with time [22]. Next, the craniotomy and durotomy is performed and the brain is exposed (duration ~30 min). Ideally, by the time of tumor resection (Fig. 4c), the conventional fast frozen pathological diagnosis has been reported to the surgeon. Using validated SRH, it will then be possible to analyze the resection borders during the intervention. In case of residual tumor, the surgeon could repeatedly add SRH results to anatomical, fluorescence [5, 23] and neurophysiological [18] criteria to decide continuation or to stop resection.

# Acknowledgements

We thank C. Ryba and J. Göldner for technical support.

# References

1. Capper D, Jones DTW, Sill M, Hovestadt V, Schrimpf D, Sturm D, Koelsche C, Sahm F, Chavez L, Reuss DE, Kratz A, Wefers AK, Huang K, Pajtler KW, Schweizer L, Stichel D, Olar A, Engel NW, Lindenberg K, Harter PN, Braczynski AK, Plate KH, Dohmen H, Garvalov BK, Coras R, Hölsken A, Hewer E, Bewerunge-Hudler M, Schick M, Fischer R, Beschorner R, Schittenhelm J, Staszewski O, Wani K, Varlet P, Pages M, Temming P, Lohmann D, Selt F, Witt H, Milde T, Witt O, Aronica E, Giangaspero F, Rushing E, Scheurlen W, Geisenberger C, Rodriguez FJ, Becker A, Preusser M, Haberler C, Bjerkvig R, Cryan J, Farrell M, Deckert M, Hench J, Frank S, Serrano J, Kannan K, Tsirigos A, Brück W, Hofer S, Brehmer S, Seiz-Rosenhagen M, Hänggi D, Hans V, Rozsnoki S, Hansford JR, Kohlhof P, Kristensen BW, Lechner M, Lopes B, Mawrin C, Ketter R, Kulozik A, Khatib Z, Heppner F, Koch A, Jouvet A, Keohane C, Mühleisen H, Mueller W, Pohl U, Prinz M, Benner A, Zapatka M, Gottardo NG, Driever PH, Kramm CM, Müller HL, Rutkowski S, von Hoff K, Frühwald MC, Gnekow A, Fleischhack G, Tippelt S, Calaminus G, Monoranu C-M, Perry A, Jones C, Jacques TS, Radlwimmer B, Gessi M, Pietsch T, Schramm J, Schackert G, Westphal M, Reifenberger G, Wesseling P, Weller M, Collins VP, Blümcke I, Bendszus M, Debus J, Huang A, Jabado N, Northcott PA, Paulus W, Gajjar A, Robinson GW, Taylor MD, Jaunmuktane Z, Ryzhova M, Platten M, Unterberg A, Wick W, Karajannis MA, Mittelbronn M, Acker T, Hartmann C, Aldape K, Schüller U, Buslei R, Lichter P, Kool M, Herold-Mende C, Ellison DW, Hasselblatt M, Snuderl M, Brandner S, Korshunov A, von Deimling A, Pfister SM (2018) DNA methylation-based classification of central nervous system tumours. Nature 555:469–474. doi: 10.1038/nature26000

2. Cohen J (1960) A coefficient of agreement for nominal scales. Educational and psychological measurement 20:37–46

3. Desroches J, Jermyn M, Mok K, Lemieux-Leduc C, Mercier J, St-Arnaud K, Urmey K, Guiot M-C, Marple E, Petrecca K (2015) Characterization of a Raman spectroscopy probe system for intraoperative brain tissue classification. Biomedical optics express 6:2380–2397

4. Eichberg DG, Shah AH, Di L, Semonche AM, Jimsheleishvili G, Luther EM, Sarkiss CA, Levi AD, Gultekin SH, Komotar RJ (2019) Stimulated Raman histology for rapid and accurate intraoperative diagnosis of CNS tumors: prospective blinded study. Journal of neurosurgery 134:137–143

5. Fountain DM, Bryant A, Barone DG, Waqar M, Hart MG, Bulbeck H, Kernohan A, Watts C, Jenkinson MD (2020) Intraoperative imaging technology to maximise extent of resection for glioma: a network meta‐analysis. Cochrane Database of Systematic Reviews

6. Freudiger CW, Min W, Saar BG, Lu S, Holtom GR, He C, Tsai JC, Kang JX, Xie XS (2008) Label-Free Biomedical Imaging with High Sensitivity by Stimulated Raman Scattering Microscopy. Science 322:1857. doi: 10.1126/science.1165758

7. Freudiger CW, Yang W, Holtom GR, Peyghambarian N, Xie XS, Kieu KQ (2014) Stimulated Raman scattering microscopy with a robust fibre laser source. Nature Photonics 8:153–159. doi: 10.1038/nphoton.2013.360

8. Höhne J, Schebesch K-M, Zoubaa S, Proescholdt M, Riemenschneider MJ, Schmidt NO (2021) Intraoperative imaging of brain tumors with fluorescein: confocal laser endomicroscopy in neurosurgery. Clinical and user experience. Neurosurgical Focus FOC 50:E19. doi: 10.3171/2020.11.FOCUS20783

9. Hollon TC, Pandian B, Adapa AR, Urias E, Save AV, Khalsa SSS, Eichberg DG, D’Amico RS, Farooq ZU, Lewis S, Petridis PD, Marie T, Shah AH, Garton HJL, Maher CO, Heth JA, McKean EL, Sullivan SE, Hervey-Jumper SL, Patil PG, Thompson BG, Sagher O, McKhann GM, Komotar RJ, Ivan ME, Snuderl M, Otten ML, Johnson TD, Sisti MB, Bruce JN, Muraszko KM, Trautman J, Freudiger CW, Canoll P, Lee H, Camelo-Piragua S, Orringer DA (2020) Near real-time intraoperative brain tumor diagnosis using stimulated Raman histology and deep neural networks. Nature Medicine 26:52–58. doi: 10.1038/s41591-019-0715-9

10. Jermyn M, Mercier J, Aubertin K, Desroches J, Urmey K, Karamchandiani J, Marple E, Guiot M-C, Leblond F, Petrecca K (2017) Highly accurate detection of cancer in situ with intraoperative, label-free, multimodal optical spectroscopy. Cancer research 77:3942–3950

11. Ji M, Lewis S, Camelo-Piragua S, Ramkissoon SH, Snuderl M, Venneti S, Fisher-Hubbard A, Garrard M, Fu D, Wang AC (2015) Detection of human brain tumor infiltration with quantitative stimulated Raman scattering microscopy. Science translational medicine 7:309ra163-309ra163

12. Landis JR, Koch GG (1977) The measurement of observer agreement for categorical data. biometrics 159–174

13. Louis DN, Wesseling P, Aldape K, Brat DJ, Capper D, Cree IA, Eberhart C, Figarella‐Branger D, Fouladi M, Fuller GN (2020) cIMPACT‐NOW update 6: new entity and diagnostic principle recommendations of the cIMPACT‐Utrecht meeting on future CNS tumor classification and grading

14. Martirosyan NL, Eschbacher JM, Kalani MYS, Turner JD, Belykh E, Spetzler RF, Nakaji P, Preul MC (2016) Prospective evaluation of the utility of intraoperative confocal laser endomicroscopy in patients with brain neoplasms using fluorescein sodium: experience with 74 cases. Neurosurgical Focus FOC 40:E11. doi: 10.3171/2016.1.FOCUS15559

15. Meyer M, Keith-Rokosh J, Reddy H, Megyesi J, Hammond RR (2010) Sources of Error in Neuropathology Intraoperative Diagnosis. Canadian Journal of Neurological Sciences / Journal Canadien des Sciences Neurologiques 37:620–624. doi: 10.1017/S0317167100010799

16. Orringer DA, Pandian B, Niknafs YS, Hollon TC, Boyle J, Lewis S, Garrard M, Hervey-Jumper SL, Garton HJL, Maher CO, Heth JA, Sagher O, Wilkinson DA, Snuderl M, Venneti S, Ramkissoon SH, McFadden KA, Fisher-Hubbard A, Lieberman AP, Johnson TD, Xie XS, Trautman JK, Freudiger CW, Camelo-Piragua S (2017) Rapid intraoperative histology of unprocessed surgical specimens via fibre-laser-based stimulated Raman scattering microscopy. Nature Biomedical Engineering 1:0027. doi: 10.1038/s41551-016-0027

17. Pekmezci M, Morshed RA, Chunduru P, Pandian B, Young J, Villanueva-Meyer JE, Tihan T, Sloan EA, Aghi MK, Molinaro AM, Berger MS, Hervey-Jumper SL (2021) Detection of glioma infiltration at the tumor margin using quantitative stimulated Raman scattering histology. Scientific Reports 11:12162. doi: 10.1038/s41598-021-91648-8

18. Raabe A, Beck J, Schucht P, Seidel K (2014) Continuous dynamic mapping of the corticospinal tract during surgery of motor eloquent brain tumors: evaluation of a new method. Journal of neurosurgery 120:1015–1024

19. Ringel F, Ingerl D, Ott S, Meyer B (2009) VARIOGUIDE: A NEW FRAMELESS IMAGE‐GUIDED STEREOTACTIC SYSTEM—ACCURACY STUDY AND CLINICAL ASSESSMENT. Operative Neurosurgery 64:ons365–ons373. doi: 10.1227/01.NEU.0000341532.15867.1C

20. Seidel K, Schucht P, Beck J, Raabe A (2020) Continuous Dynamic Mapping to Identify the Corticospinal Tract in Motor Eloquent Brain Tumors: An Update. J Neurol Surg A Cent Eur Neurosurg 81:105–110

21. Senft C, Bink A, Franz K, Vatter H, Gasser T, Seifert V (2011) Intraoperative MRI guidance and extent of resection in glioma surgery: a randomised, controlled trial. The lancet oncology 12:997–1003

22. Stieglitz LH, Fichtner J, Andres R, Schucht P, Krähenbühl A-K, Raabe A, Beck J (2013) The silent loss of neuronavigation accuracy: a systematic retrospective analysis of factors influencing the mismatch of frameless stereotactic systems in cranial neurosurgery. Neurosurgery 72:796–807

23. Stummer W, Pichlmeier U, Meinel T, Wiestler OD, Zanella F, Reulen H-J (2006) Fluorescence-guided surgery with 5-aminolevulinic acid for resection of malignant glioma: a randomised controlled multicentre phase III trial. The Lancet Oncology 7:392–401. doi: 10.1016/S1470-2045(06)70665-9

24. Tofte K, Berger C, Torp SH, Solheim O (2014) The diagnostic properties of frozen sections in suspected intracranial tumors: A study of 578 consecutive cases. Surgical neurology international 5

25. Uematsu Y, Owai Y, Okita R, Tanaka Y, Itakura T (2007) The usefulness and problem of intraoperative rapid diagnosis in surgical neuropathology. Brain tumor pathology 24:47–52

# Figure Legends


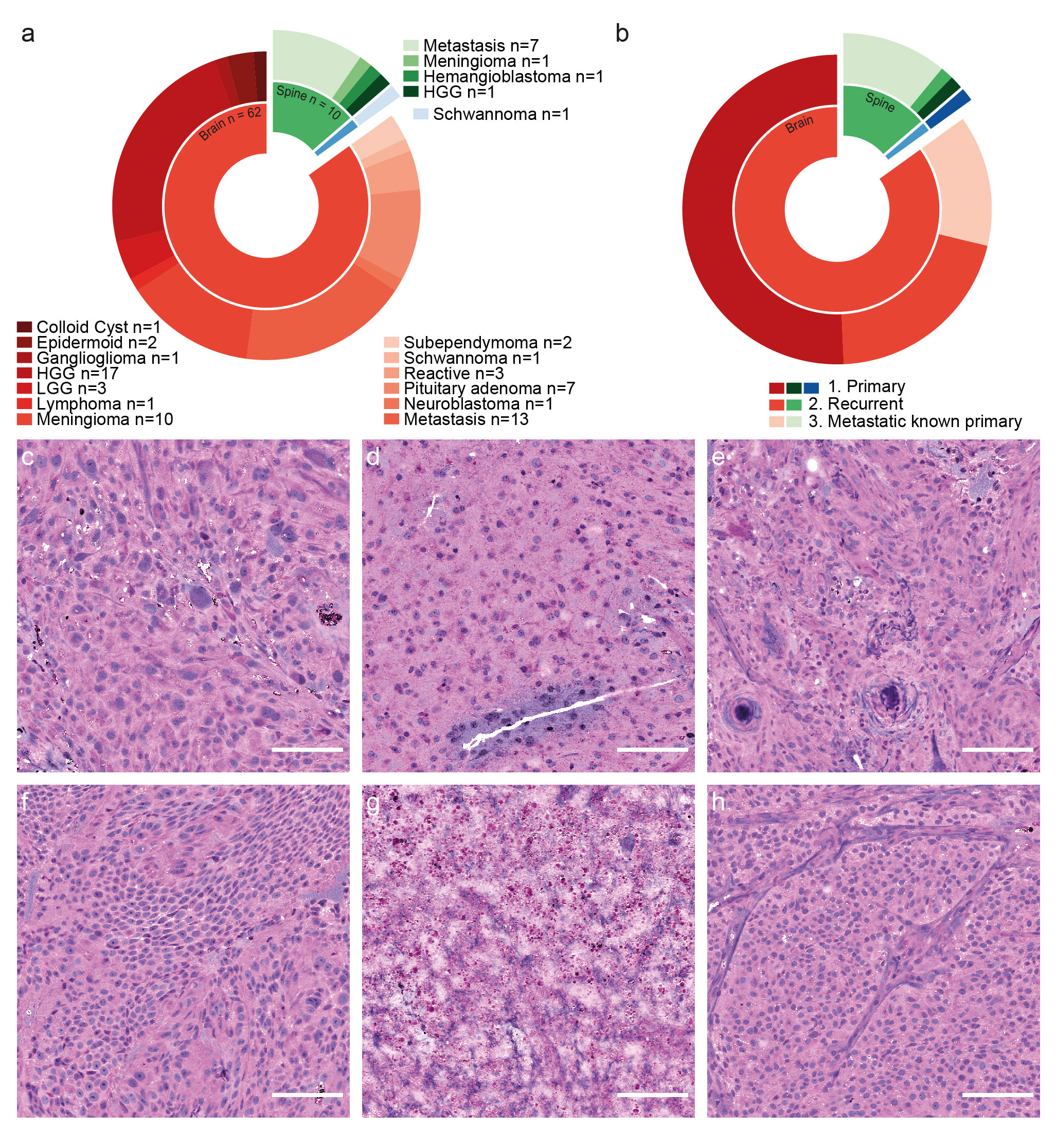


**Fig. 1 Data and Stimulated Raman Histology**

**a** Category and location of 73 cranial, spinal and peripheral (blue) tumors. **b** Distribution of patient history **c-h** Illustrative examples of SRH images. **c** a GBM of the left parietal lobe in 72 y/o female. **d** 53 y/o. male with left frontal oligodendroglioma WHO grade II. **e** Spinal (TH 2/3) psammomatous meningioma in a 78 y/o male. **f** Left frontal dural metastasis of an esophageal cancer in a 65 y/o male. **g** Reactive gliosis with necrotic components (shown) after radiation of a left temporo-occipital melanoma metastasis in a 40 y/o female. **h** Non hormone active pituitary adenoma in a 56 y/o male. Scale bars: 100 µm

Program used to create figure: Adobe Illustrator CS 6


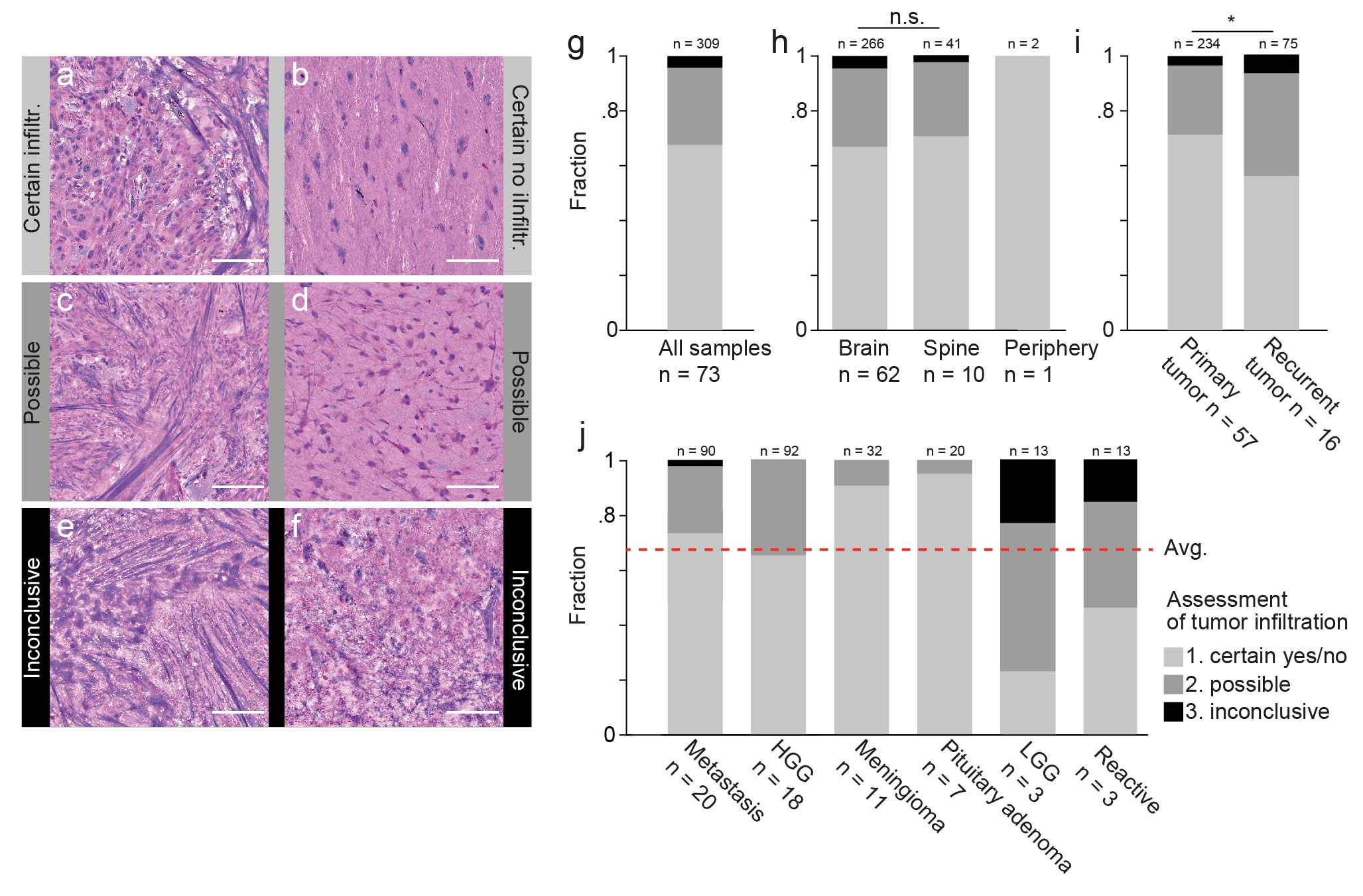


**Fig. 2 Assessment of tumor infiltration using SRH imaging**

**a-f** Examples of subjective classification of tumor infiltration in SRH images as certain (a,b), possible (c,d) and inconclusive (e,f): **a** Certain tumor infiltration in case of 78 y/o female with metastasis of NSCLC in the right frontal lobe. **b** Certain absence of tumor infiltration in case of cortical access tissue for resection of a right temporo-occipital GBM. **c** 72 y/o male patient with spinal metastasis of laryngeal squamous cell carcinoma. **d** 49 y/o male with recurrent left frontal GBM. **e** 77 y/o female with recurrent left temporal NSCLC metastasis. **f** 40 y/o female with metastasis of malignant melanoma in the left temporo-occipital lobe. **g** Overall assessment of tumor infiltration in 309 SRH images from 73 neurosurgical cases (cf. Fig. 1a). **h** Stratification of assessment of tumor infiltration according to tumor location and **i** the medical history. **j** Stratification of assessment of tumor infiltration according to diagnostic category (cf. Fig 1a). Shown here are the 6 categories that contained >3 cases and >10 SRH images per category. Red line shows overall average (cf. Fig. 2g). Above all bars are the number of SRH images; below the number of cases per category. Scale bars: 100 µm

Program used to create figure: Adobe Illustrator CS 6


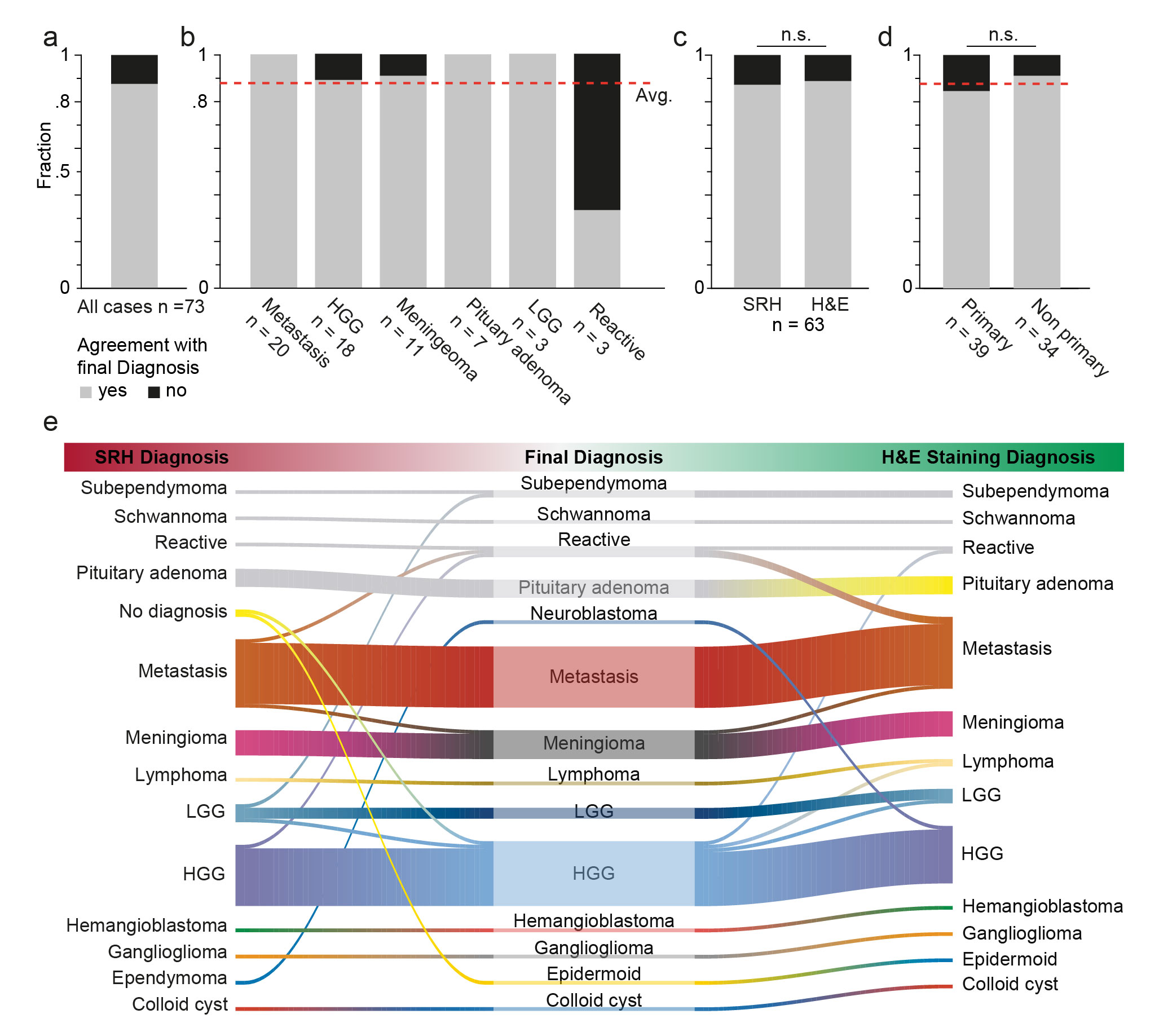


**Fig. 3 Accuracy of diagnosis based on medical history and SRH images**

A board certified neuropathologist novice in the assessment of SRH images stated a diagnosis based on the SRH images and the clinical information in 73 cases of cranial, spinal or peripheral tumors. **a** Overall agreement of the diagnosis compared to the final neuropathological diagnosis was 87.7% (cf. red line in b and d). **b** Stratification of diagnostic accuracy according to tumor entity. Shown here are the 6 entities that contained >3 cases. Below all bars are the number of patients per category. **c** Non-inferiority of diagnostic accuracy of SRH vs. conventional fast frozen section using H&E staining (87.3 vs. 88.9%, p=0.783 chi-squared). **d** Non-significant lower accuracy in primary tumor cases (cf. Group 1 Fig. 1b) vs. non-primary cases (cf. Group 2 and 3 Fig. 1b) (84.6 vs. 91.2%, respectively; p=0.395 chi-squared). **e** River plot showing the correspondence of SRH based diagnosis (left) and H&E stained fast frozen sections (right) to the definitive neuropathological diagnosis (middle) with misclassifications appearing as lane changes

Program used to create figure: Adobe Illustrator CS 6


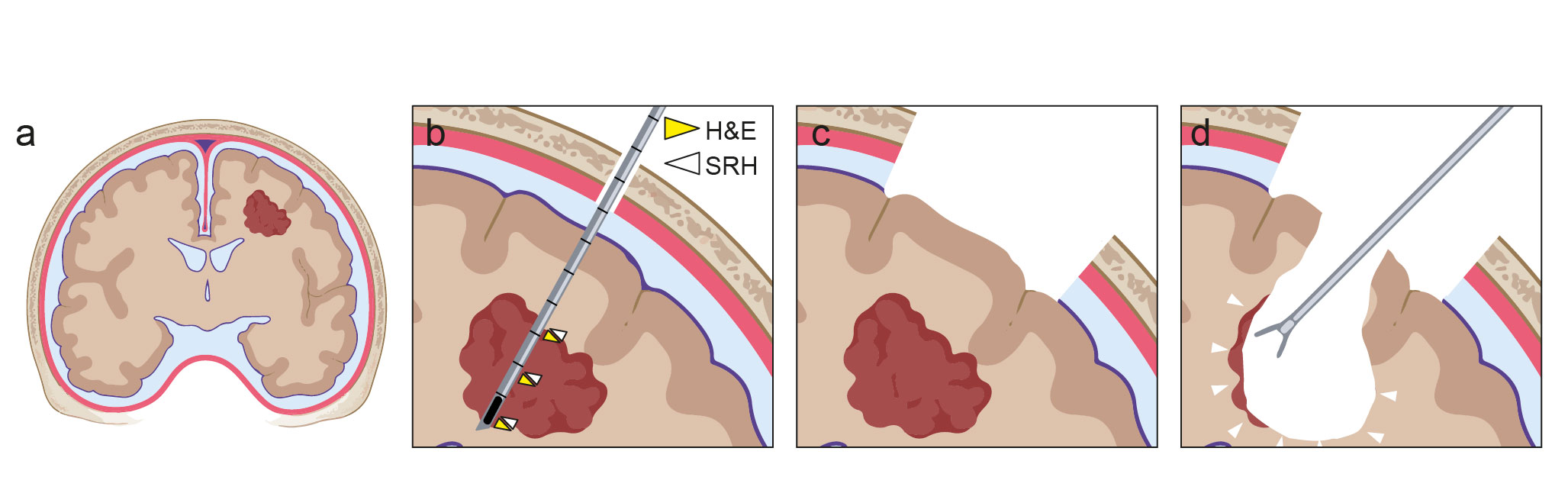


**Fig. 4 Workflow for the exploration of histopathological-based neurosurgical decision-making**

**a** Coronal schematic of possible neurosurgical approach for brain tumors using intraoperative SRH imaging for detection of the tumor borders. **b** Use of a frameless image-guided stereotactic biopsy system through a burr hole at the center of the planned craniotomy. Yellow arrowhead symbolize samples dedicated to conventional neuropathological diagnostic and white arrowhead symbolize intraoperative SRH. **c** Craniotomy with potential brain shift and loss of navigation accuracy over time. **d** Sampling of resection borders to guide resection or for assessment of final resection margins.

Program used to create figure: Adobe Illustrator CS 6 and BioRender.com (2021).


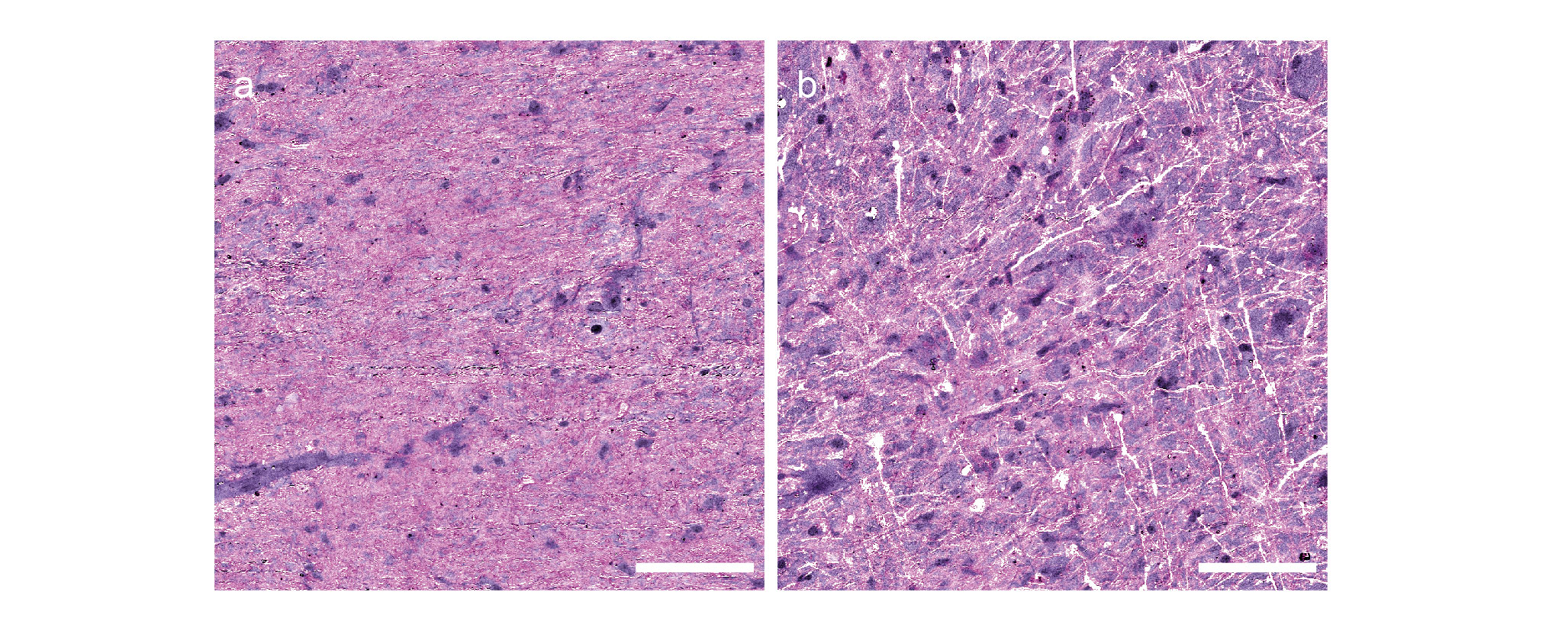


**Suppl. Fig. 1 White matter phenotypes in SRH**

**a** Example of a putatively healthy white matter vs. **b** infiltrated white matter in case of GBM. Note the appearance of white fibers, presumably axons in the infiltrated white matter, which are not visible in the non-infiltrated case.
